# Cyclic di‐GMP inactivates T6SS and T4SS activity in *Agrobacterium tumefaciens*

**DOI:** 10.1111/mmi.14279

**Published:** 2019-06-04

**Authors:** Ronan R. McCarthy, Manda Yu, Kira Eilers, Yi‐Chieh Wang, Erh‐Min Lai, Alain Filloux

**Affiliations:** ^1^ MRC Centre for Molecular Bacteriology and Infection, Department of Life Sciences Imperial College London London SW7 2AZ UK; ^2^ Division of Biosciences, Department of Life Sciences College of Health and Life Sciences, Brunel University London Uxbridge UB8 3PH UK; ^3^ Institute of Plant and Microbial Biology Academia Sinica Taipei 11529 Taiwan

## Abstract

The Type VI secretion system (T6SS) is a bacterial nanomachine that delivers effector proteins into prokaryotic and eukaryotic preys. This secretion system has emerged as a key player in regulating the microbial diversity in a population. In the plant pathogen *Agrobacterium tumefaciens*, the signalling cascades regulating the activity of this secretion system are poorly understood. Here, we outline how the universal eubacterial second messenger cyclic di‐GMP impacts the production of T6SS toxins and T6SS structural components. We demonstrate that this has a significant impact on the ability of the phytopathogen to compete with other bacterial species *in vitro* and *in planta*. Our results suggest that, as opposed to other bacteria, c‐di‐GMP turns down the T6SS in *A. tumefaciens* thus impacting its ability to compete with other bacterial species within the rhizosphere. We also demonstrate that elevated levels of c‐di‐GMP within the cell decrease the activity of the Type IV secretion system (T4SS) and subsequently the capacity of *A. tumefaciens* to transform plant cells. We propose that such peculiar control reflects on c‐di‐GMP being a key second messenger that silences energy‐costing systems during early colonization phase and biofilm formation, while low c‐di‐GMP levels unleash T6SS and T4SS to advance plant colonization.

## Introduction

Interspecies bacterial competition plays a key role in shaping microbial populations and determining what bacterial species are dominant in a given niche (Kapitein and Mogk, 2014; Bernal *et al.*, 2017a; McNally *et al.*, 2017; Chassaing and Cascales, 2018). It is indeed increasingly clear that any specific environmental niche, including within various parts of the human body (Rojo *et al.*, 2017), will profile the establishment of specific microbial populations. How bacteria respond to environmental variations or intrusion of ‘foreign organisms’ by triggering an attack/defence war game with other bacteria is not clearly understood. Yet, despite the numerous protein secretion systems encoded within bacterial genomes, one has emerged as being the ‘weapon’ of choice through which bacteria mediate interspecies competition, for example, in the human gut (Russell *et al.*, 2014b; Chatzidaki‐Livanis *et al.*, 2016; Sana *et al.*, 2016; Anderson *et al.*, 2017) or *in planta* (Ma *et al.*, 2014; Bernal *et al.*, 2017a; 2017b). This system, termed the Type VI Secretion System (T6SS), is found on the genome of a wide variety of Gram‐negative bacteria and can deliver a remarkable array of toxins such as nucleases, amidases and phospholipases (Russell *et al.*, 2011; Russell *et al.*, 2012; Ma *et al.*, 2014; Russell *et al.*, 2014a; Alcoforado Diniz *et al.*, 2015). It is noticeable that in Gram‐positive bacteria the type VII secretion system (T7SS) is now emerging as the antibacterial nano‐weapon (Cao *et al.*, 2016). Whereas many of these toxins are antibacterial (Russell *et al.*, 2014a), some are also designed to target eukaryotic host cells (Hachani *et al.*, 2016) and fungi (Trunk *et al.*, 2018) or have a dual function like phospholipases (Jiang *et al.*, 2014). These toxins can be encoded as part of a large T6SS cluster and thus genetically linked with genes encoding core T6SS components, or be found independently in so‐called *vgrG/hcp* islands (Hachani *et al.*, 2014; Whitney *et al.*, 2014). It has recently emerged that T6SS toxins can specifically interact with VgrG proteins (Bondage *et al.*, 2016; Cianfanelli *et al.*, 2016a; Flaugnatti *et al.*, 2016), the puncturing device of the T6SS nanomachine, or can be found fused to VgrG thus forming what is referred to as an evolved VgrG (Pukatzki *et al.*, 2007; Durand *et al.*, 2012) or fused to a PAAR domain which loads the toxin onto a cognate VgrG (Whitney *et al.*, 2015; Quentin *et al.*, 2018). Some bacterial species have been shown to encode numerous T6SSs, with experimental evidence highlighting specific target or prey such as the H1‐T6SS in *Pseudomonas aeruginosa* being associated with interbacterial competition while the H2‐T6SS was proposed to preferentially target eukaryotic cells (Sana *et al.*, 2012; Hachani *et al.*, 2014; Jones *et al.*, 2014). Now, it seems that a clear‐cut system specificity is unlikely to be the case as the H2‐T6SS was recently shown to also be antibacterial (Allsopp *et al.*, 2017).

**Figure 1:**
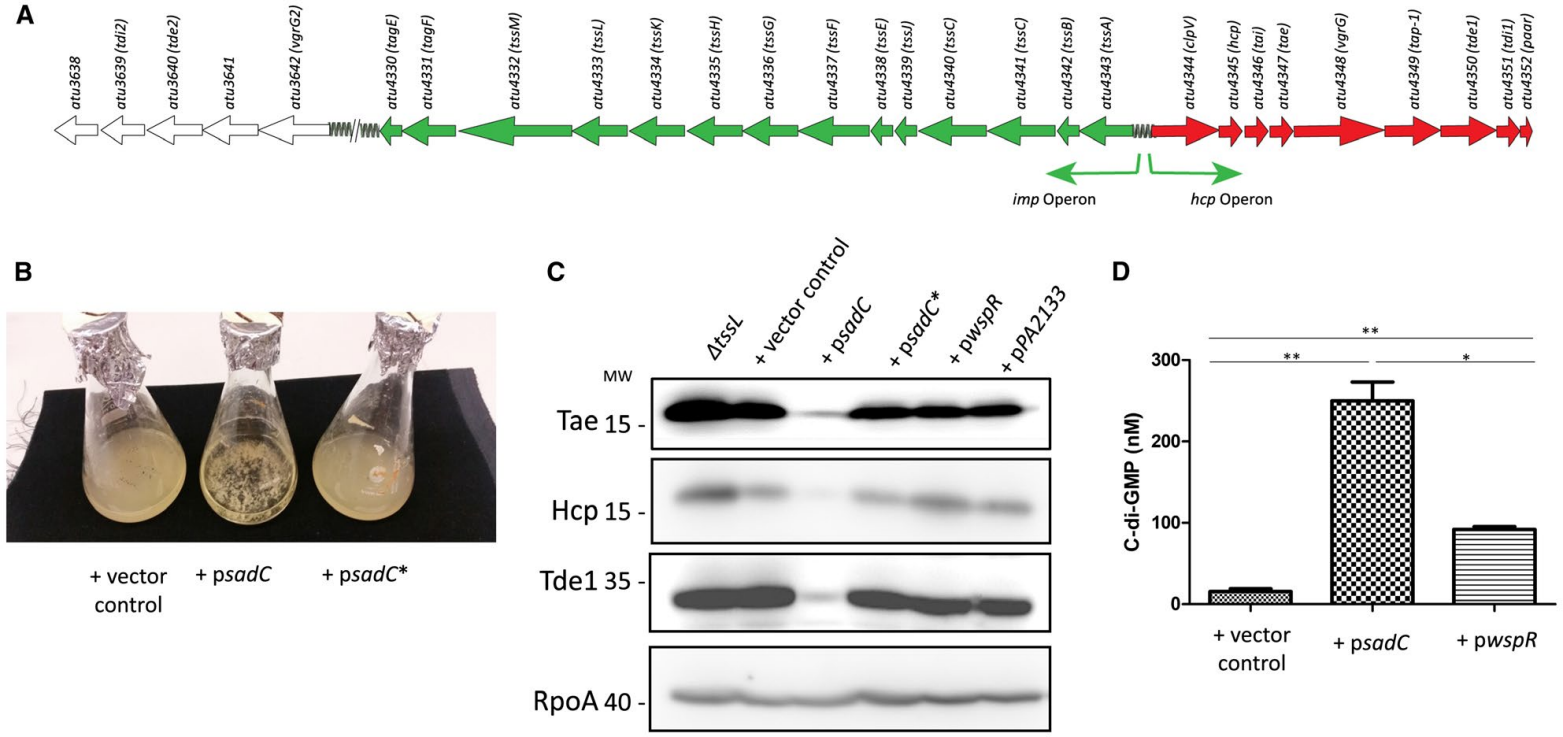
Impact of c‐d‐GMP on Tde1, Tae and Hcp levels. A. The T6SS cluster composed of the divergently transcribed *imp* (Green) and *hcp* (Red) operons encoding 14 and 9 genes, respectively, and the remote island starting from *atu3642* (vgrG‐2) encoded by *A. tumefaciens* strain C58. B. Expression of wild‐type SadC and a catalytically inactive SadC (psadC*) in wild‐type *A. tumefaciens* grown to early exponential phase in 523 medium. C. Western blots using Tde1, Tae, Hcp and RpoA (Loading Control) antibodies on whole lysates of Δ*tssL* cells (No secretion control) or wild‐type cells transformed with either pBBRMCS4 (vector control), p*sadC*, psadC*, p*wspR* and p*PA2133* grown to early exponential phase in 523 medium. D. C‐di‐GMP levels were quantified via LC‐MS/MS. Transformed cells were grown for 16 h in 523 medium and cells equivalent to an OD600 5 were collected. Samples of interest were compared to a standard curve derived from measurements of known concentrations of pure c‐di‐GMP to determine the concentration (in nM) of c‐di‐GMP in the samples. All experiments are the mean of two independent biological experiments with standard deviation error bars. Statistical significance was determined using students *t*‐test with p < 0.05*, p < 0.01 **, p < 0.001***.

*Agrobacterium tumefaciens* is a soil bacterium that causes crown gall disease in a wide range of plants as a result of the delivery of T‐DNA to plant cells via the Type IV Secretion System (T4SS) (Pitzschke and Hirt, 2010; Christie *et al.*, 2014; Hwang *et al.*, 2017). *Agrobacterium tumefaciens* strain C58 has also been shown to carry a single T6SS cluster composed of two divergently transcribed operons and featuring three toxins, Tae, a putative peptidoglycan amidase, and two DNases, Tde1 and Tde2 (Fig. 1A) (Ma *et al.*, 2014). The Tde effectors have been shown to play a role in interbacterial competition and plant colonization. While ample information is now available consistently describing the structural components of the T6SS nanomachine (Cianfanelli *et al.*, 2016b; Joshi *et al.*, 2017; Nguyen *et al.*, 2018) and its associated arsenal of toxins (Durand *et al.*, 2014; Hachani *et al.*, 2016; Lien and Lai, 2017), the type of regulatory elements that control expression of the system is variable from one species to another and has not always been conclusively determined. Well characterized signalling cascades have been shown to play a role in activating the T6SS such as the Gac/Rsm cascade (Moscoso *et al.*, 2011; Allsopp *et al.*, 2017) and the LasR/MvfR (PqsR) quorum sensing systems in *P. aeruginosa* (Lesic *et al.*, 2009; Majerczyk *et al*., 2016; Sana *et al.*, 2012), or, for example, the autoinducer‐2 quorum sensing system and QstR in *Vibrio cholerae* (Ishikawa *et al.*, 2009; Jaskolska *et al.*, 2018). The environmental stimuli that may trigger T6SS expression are also poorly understood but some modulators have been proposed to be bile salts (Reen *et al.*, 2012; Bachmann *et al.*, 2015), contact dependent and counter‐attack based induction (Russell *et al.*, 2011; Basler *et al.*, 2013), part of the competence regulon in *V. cholerae* (Borgeaud *et al.*, 2015), or danger signal released from lysing cells in a *P. aeruginosa* population (LeRoux *et al.*, 2015). To date in *A. tumefaciens* C58, only the ExoR/ChvG/ChvI signalling cascade has been shown to be capable of regulating the T6SS, with induction of this system at low environmental pH leading to activation of the T6SS (Wu *et al.*, 2012). This is believed to play a key role in allowing *A. tumefaciens* dominate around the site of a wound in a plant, as low pH is characteristic of this niche (Wu *et al.*, 2012; Heckel *et al.*, 2014).

Response to external stimuli can be further driven by intracellular second messengers and this is the case for the universal eubacterial second messenger molecule cyclic di‐GMP (c‐di‐GMP) (Jenal *et al.*, 2017), which is tightly associated with biofilm development in a number of bacterial species including *P. aeruginosa* and *A. tumefaciens* (Heindl *et al.*, 2014; Valentini and Filloux, 2016). The c‐di‐GMP signalling network can impact virulence in both human and plant pathogens as, for example, seen with the DgcP cyclase in controlling infections by *P. aeruginosa* or *Pseudomonas savastanoi pv savastanoi*, an olive tree pathogen (Aragon *et al.*, 2015). Remarkably, in *P. aeruginosa*, c‐di‐GMP signalling has been shown to regulate T6SS, but while high levels correlate with increased levels in biofilm formation and T6SS activity (Moscoso *et al.*, 2011), low levels of this second messenger are associated with increased motility and Type III secretion system (T3SS) activity (Moscoso *et al.*, 2011). The biofilm part of this paradigm has been confirmed within *A. tumefaciens*. Indeed, one of the pioneering studies on c‐di‐GMP as a signalling molecule has been carried out in this organism, where it was shown that the activity of a cellulose synthase was dependent on the binding of c‐di‐GMP (Amikam and Benziman, 1989). Subsequent studies have demonstrated the ability of high levels of c‐di‐GMP to increase the levels of a number of different *A. tumefaciens* polysaccharides and as a result biofilm formation (Heindl *et al.*, 2014; Feirer *et al.*, 2015). In the present study, we thus investigate the role of c‐di‐GMP in regulating the T6SS in *A. tumefaciens* C58 using a non‐native diguanylate cyclase (DGC) from *P. aeruginosa* as well as native *A. tumefaciens* DGCs. We discover that contrary to the paradigm established in *P. aeruginosa*, high levels of c‐di‐GMP represses the T6SS activity in *A. tumefaciens*. We show that this occurs at the transcriptional level and has a significant impact on the ability of *A. tumefaciens* to attack and outcompete other bacterial species. We also demonstrate that this is not specific to a single *A. tumefaciens* DGC but that several of these enzymes can have a significant impact. Furthermore, we show that elevated levels of c‐di‐GMP within the cell also impact the expression of key structural components of the T4SS. This transcriptional influence subsequently impacts the capacity of *A. tumefaciens* to transform plant cells as determined by a transient transformation assay. This duality is quite remarkable and suggests that at a specific stage during the *A. tumefaciens* colonization process interaction with the rhizosphere and with the plant cells should both be kept silent.

## Results

### The *P. aeruginosa* diguanylate cyclase SadC supresses T6SS activity in *A. tumefaciens* C58

The universal second messenger c‐di‐GMP regulates a wide variety of bacterial phenotypes including motility, protein secretion, exopolysaccharide production, virulence, or biofilm formation (Romling and Balsalobre, 2012; Romling *et al.*, 2013; Jenal *et al.*, 2017). Across almost all eubacteria, paradigms have emerged that correlate high intracellular levels of c‐di‐GMP with loss of motility, increased polysaccharide production and biofilm formation (Valentini and Filloux, 2016; Conner *et al.*, 2017). The T6SS has also emerged as being subject to c‐di‐GMP control, with high levels of c‐di‐GMP associated with activation and low levels associated with suppression of this secretion system (Moscoso *et al.*, 2011; Romling *et al.*, 2013) as shown clearly with *P. aeruginosa* and to a lesser extent with *Vibrio alginolyticus*. In the latter case, it was proposed that the PppA phosphatase has a negative impact on both the activity of the T6SS and the levels of c‐di‐GMP (Sheng *et al.*, 2013). In the case of *Pseudomonas fluorescens* it was also proposed that c‐di‐GMP could directly bind to the ClpB2 ATPase one of the core component in the T6SS (Trampari *et al.*, 2015). In *A. tumefaciens* C58, it has been shown that high levels of c‐di‐GMP are associated with increased extracellular polysaccharide (EPS) production and biofilm formation (Heindl *et al.*, 2014; Feirer *et al.*, 2015), but the impact of c‐di‐GMP on T6SS has not been explored. *Agrobacterium tumefaciens* has one T6SS cluster which is composed of two divergently transcribed operons, the *imp* operon which primarily encodes the structural components of the secretion system and the *hcp* operon that encodes two of the known *A. tumefaciens* T6SS toxins, i.e. Tae and Tde1 (Wu *et al.*, 2008; Lin *et al.*, 2014). There is also an orphan cluster that encodes Tde2, the only known T6SS toxin not directly encoded in the *A. tumefaciens* primary cluster (Fig. 1A) (Ma *et al.*, 2014). Here, we investigate the impact of c‐di‐GMP on the functionality of this system, and introduced plasmids encoding the well characterized *P. aeruginosa* diguanylate cyclases (DGC) SadC and WspR (Guvener and Harwood, 2007; Merritt *et al.*, 2007a; Dahlstrom and O'Toole, 2017; McCarthy *et al.*, 2017a), a mutated version of SadC that has the canonical catalytic site GGEEF domain changed to AAAEF (psadC*) and a known functional phosphodiesterase PA2133 (Ueda and Wood, 2010), into a wild‐type strain of *A. tumefaciens*. The recombinant strains were grown to early exponential phase and the levels of the T6SS DNase effector Tde1, amidase effector Tae and T6SS nanotube building block Hcp were assessed by western blotting (Ma *et al.*, 2014). Firstly, it was striking to observe that the clumping/hyperbiofilm phenotype resulting from SadC overexpression and usually associated with high levels of c‐di‐GMP (Moscoso *et al.*, 2014) was no longer seen when the catalytically inactive sadC* mutant was expressed indicating as expected that this enzyme was no longer capable of generating c‐di‐GMP (Fig. 1B). The reduction in activity of this mutated version of SadC was confirmed by LC‐MS/MS and was shown to be comparable to another mutated version of SadC that had the GGEEF domain changed to GGAAF (Supporting Information Fig. S1). Furthermore, it was readily observed that expression of the *sadC*‐encoded membrane bound DGC dramatically abrogated Tde1, Tae and Hcp production. Levels seen in the strains containing plasmids encoding the SadC catalytic site mutant were comparable to strains carrying the empty vector (pBBRMCS4) (Fig. 1C) suggesting that the intracellular levels of c‐di‐GMP were effectively responsible for the reduction in the level of these T6SS components and toxins. The expression of the *wspR*‐encoded cytoplasmic DGC did not alter the levels of Tde1, Tae or Hcp (Fig. 1C). This suggests that the intracellular activity of WspR in *A. tumefaciens* might not be strong enough to impact the levels of Tde1/Hcp or that membrane association of the DGC is instrumental to the phenotype. Note that the overexpression of the cytoplasmic phosphodiesterase (PDE) PA2133 also did not significantly impact the levels of Tde1, Tae or Hcp (Fig. 1C). The proposed reduced activity of WspR in *A. tumefaciens* was confirmed by quantifying the levels of c‐di‐GMP using LC‐MS/MS. This analysis confirmed that SadC produced significantly more c‐di‐GMP than WspR when expressed in *A. tumefaciens* (Fig. 1D). Overall, these findings are opposite to the established *P. aeruginosa* paradigm of positive regulation of the T6SS by c‐di‐GMP. Such variation in control is likely depending on how and where c‐di‐GMP is acting and this may dramatically differ from one bacterial species to the other. For example, in *P. aeruginosa*, the control exerted by master regulators such as RetS/LadS is observable on all H1‐T6SS genes (Allsopp *et al.*, 2017) whereas in *Pseudomonas syringae* it can only be seen on a subset of T6SS genes (e.g. *icmF*) but not on others (e.g. *hcp*) (Records and Gross, 2010).

### SadC has a significant impact on T6SS‐dependent interbacterial competition *in vitro* and *in planta*

**Figure 2:**
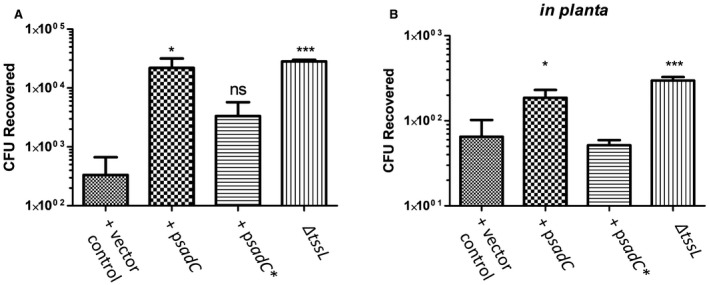
Impact of c‐di‐GMP on the ability of *A. tumefaciens* to kill other bacteria. A. *A. tumefaciens* strains with either an empty vector control, a plasmid expressing wild‐type SadC or a plasmid expressing a catalytically inactive SadC (psadC*) were incubated for 14 h on I medium with of *E. coli* DH10B containing pRL662 which harbours a gentamicin cassette. Bacteria were then resuspended in PBS and plated on LB agar containing gentamicin. B. The bacterial strains described above were resuspended in 1/2 Murashige and Skoog (MS) medium (pH 5.5) and immediately injected into the leaves of 6–8‐day‐old *N. benthamiana* plants. After 14 h incubation, coupons were cut from these leaves, homogenized in PBS and plated on LB supplemented with gentamicin (50 µg ml^−1^). All experiments are the mean of three independent biological experiments with standard deviation error bars. Statistical significance was determined using students *t*‐test comparing each strain to the vector control with p < 0.05 *, p < 0.01 **, p < 0.001***.

The T6SS is known to play a key role in shaping the microbiota and this is particularly true in the plant rhizosphere (Bernal *et al.*, 2017a; Bernal *et al.*, 2017b). To investigate if the impact of c‐di‐GMP on T6SS was biologically significant and capable of influencing the structure of a bacterial population, bacterial competition assays were performed as described in experimental procedures and using as an attacker strain *A. tumefaciens* which constitutively expresses wild‐type (p*sadC*) or mutant version of *sadC* (psadC*). The bacterial prey *Escherichia coli* carries the pRL662 plasmid conferring gentamicin resistance and survivors after *A. tumefaciens* contact could be recovered as colony forming units (CFU) on gentamicin‐containing plates. Expression of wild‐type *sadC* in *A. tumefaciens* significantly reduced the ability of *A. tumefaciens* to kill the prey bacterium *E. coli* (Fig. 2A) when compared to a strain harbouring the vector control or expressing the non‐functional version of *sadC*, sadC*. The low levels of SadC‐dependent killing were comparable to those seen with an *A. tumefaciens* mutant strain lacking a core gene for the T6SS machinery, *tssL*, thus resulting in T6SS inactivation and confirming the T6SS downregulation through SadC activity. To fully rule out that the observed reduction in killing was not a consequence of the increased cell to cell aggregation seen when overexpressing p*sadC*, the assay was repeated in *P. aeruginosa*, where increased expression of *sadC* is also known to impact cell to cell aggregation (Moscoso *et al.*, 2014). Competition assays between *P. aeruginosa* PAO1 expressing *sadC* and *E. coli* demonstrated increased levels of killing, contrary to what was observed in the *A. tumefaciens* competition assays (Supporting Information Fig. S2). A similar increase in killing was seen when using a *P. aeruginosa* Δ*rsmA* mutant, which is known to have elevated levels of *sadC* expression (Moscoso *et al.*, 2014). This suggests that the impact seen on cell‐to‐cell aggregation by the expression of *sadC* does not impact the activity of the T6SS.

To add further biological significance to this observation and partially recreate a physiologically relevant environment, the competition assays were repeated but bacteria were infiltrated in the leaves of the *Nicotiana benthamiana* plant as previously described (Ma *et al.*, 2014). This assay more or less phenocopies our *in vitro* findings with expression of *sadC* leading to significant reduction in prey killing, and thus higher CFU recovery from the plant leaves of gentamicin resistant *E. coli* cells (Fig. 2B). These findings demonstrate that the intracellular levels of c‐di‐GMP can have a significant impact on *A. tumefaciens* ability to engage *via* the T6SS in interspecies competition *in planta*.

### Endogenous *A. tumefaciens* DGCs suppress the T6SS activity

**Figure 3:**
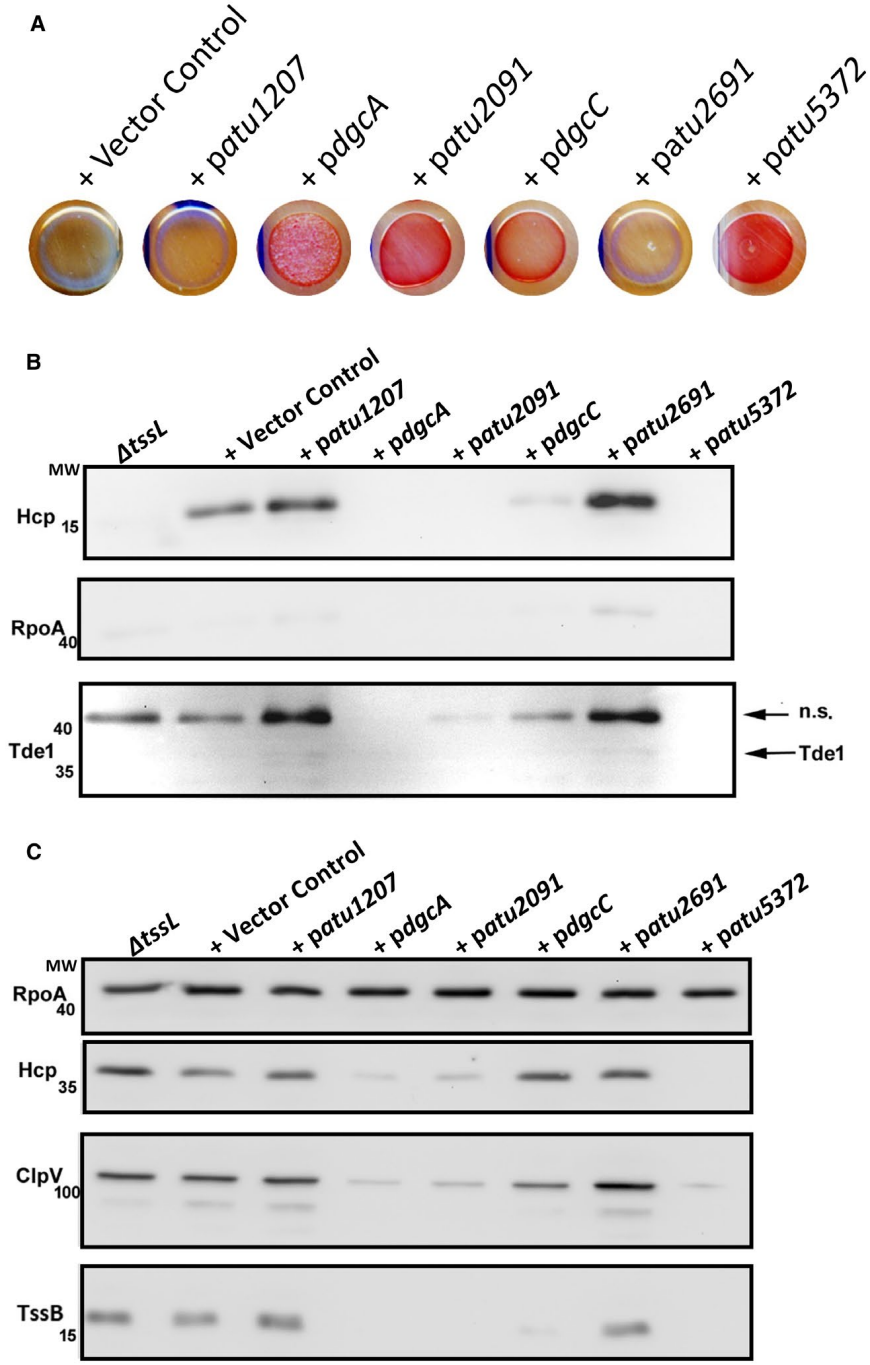
Impact of native DGCs on T6SS. A. Expression of a range of six different DGCs whose genes were cloned into wild‐type *A. tumefaciens* and spotted onto Congo red agar containing 0.5 mM IPTG and Spectinomycin 200 µg ml^−1^. B. Western blot using Hcp, Tde1, and RpoA (Loading Control) antibodies on supernatants of cells transformed with either empty vector control, p*dgcA*, p*atu2091*, p*dgcC*, p*atu2691*, p*atu1207* or p*atu5372* from cells grown to early exponential phase in 523 medium. C. Western blot using Hcp, ClpV, TssB, Tde1 and RpoA (Loading Control) antibodies on whole cell lysates of cells transformed with either empty vector control, p*dgcA*, p*atu2091*, p*dgcC*, p*atu2691*, p*atu1207* or p*atu5372* from cells grown to early exponential phase in 523 medium.

The use of a well‐characterized *P. aeruginosa* DGC to increase the intracellular levels of c‐di‐GMP in *A. tumefaciens* does not guarantee that DGCs encoded within the *A. tumefaciens* genome can have a similar impact on T6SS. To investigate this, we focused on endogenous *A. tumefaciens* DGCs, 16 of which are encoded within the genome (Romling *et al.*, 2013), of these we selected 6 that encoded transmembrane domains and carry the canonical GGDEF domain, namely Atu2091, Atu2691, Atu1207 and Atu5372 as well as the two previously described DGCs DgcA (Atu1257) and DgcC (Atu2179) both of which have been shown to impact EPS production (Xu *et al.*, 2013) (Supporting Information Fig. S3). The rationale here was that both SadC and WspR have been shown to be active DGCs, but only the membrane bound SadC was capable of impacting c‐di‐GMP associated phenotypes (Fig. 1C). This could suggest that the localization of the the DGC may have a critical impact on phenotypic outcomes. The genes encoding these DGCs were amplified and cloned under the control of the IPTG‐inducible *lac* promoter in the pTrc200 vector. Using agar plates‐containing Congo red, it is possible to reveal exopolysaccharide (EPS) production in response of increased intracellular levels of c‐di‐GMP (Howie and Brewer, 2009; Heckel *et al.*, 2014; Heindl *et al.*, 2014; Feirer *et al.*, 2015; Feirer *et al.*, 2017). It was clear that some of the *A. tumefaciens* transformed with the above recombinant plasmids yielded colonies displaying a wrinkly phenotype (Fig. 3A). This confirmed that as previously described DgcA and DgcC could impact EPS production (Xu *et al.*, 2013) but also that Atu2091 and Atu5372 were increasing the levels of exopolysaccharide production, suggesting they are active cyclases, while Atu1207 and Atu2691 had little or no effect (Fig. 3A).

To assess if anyone of these DGCs was capable of shutting down the T6SS, as SadC did, a secretion assay was performed using a Hcp and a Tde1 specific antibody as described above. This assay revealed that Atu2091, Atu5372 and DgcA were all capable of down‐regulating the secretion of Hcp and Tde1 to non‐detectable levels, and these levels were comparable to those observed with the non‐functional T6SS mutant *A. tumefaciens ΔtssL* (Fig. 3B). DgcC also had a negative impact on Hcp levels but to a lesser extent. The cellular levels of Hcp, TssB (subunit of the T6SS sheath) and ClpV (T6SS AAA^+^ ATPase), revealed a similar profile to what was seen with Hcp secretion, with expression of Atu2091, Atu5372 and DgcA leading to reduced levels of these proteins and DgcC having a lesser effect (Fig. 3C). As for the EPS assay, Atu1207 and Atu2691, did not shut off T6SS activity, thus suggesting that they may not be active or are generating levels of c‐di‐GMP too low to trigger observable phenotypic changes.

**Figure 4:**
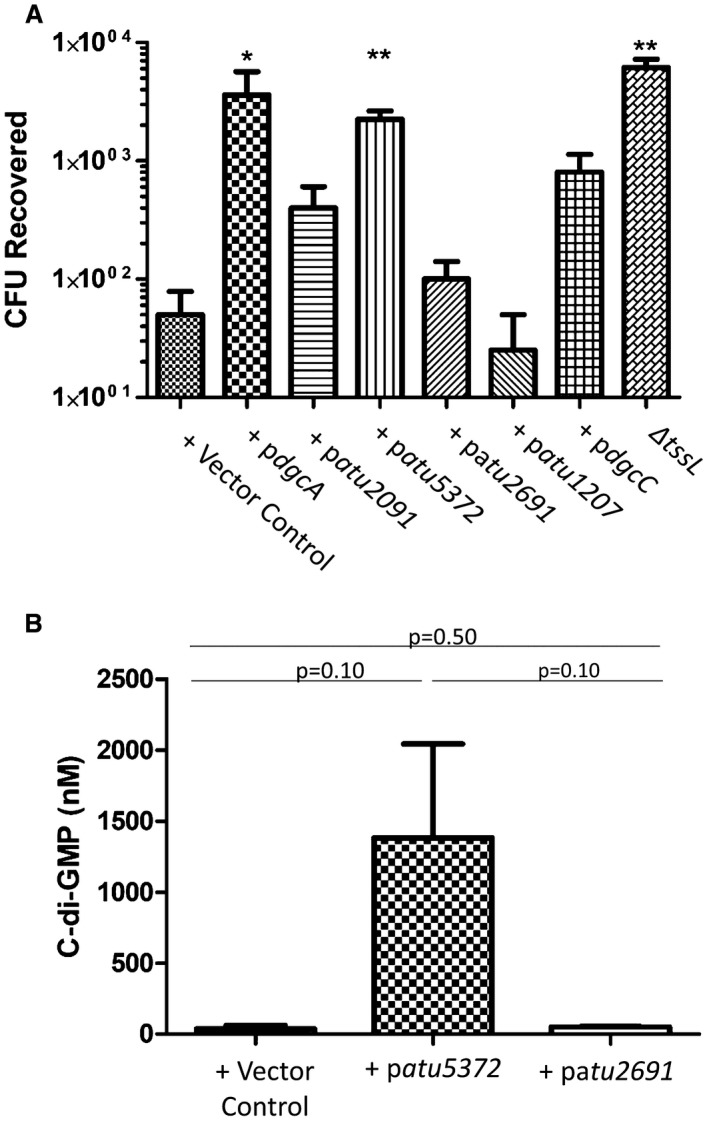
Impact of native DGC on interspecies killing and cdi‐GMP levels. A. *A. tumefaciens* strains with either an empty vector control, p*dgcA*, p*atu2091*, p*dgcC*, p*atu2691*, p*atu1207* and p*atu5372* were incubated for 14 h on a I medium (pH5.5) with *E. coli* containing pRL662 which harbours a gentamicin cassette. Bacteria were then resuspended in PBS and plated on LB agar‐containing gentamicin. All experiments are the mean of three independent biological experiments with standard deviation error bars. Statistical significance was determined using students *t*‐test comparing each strain with the vector control, p < 0.05 *, p < 0.01 ** B. C‐di‐GMP levels were quantified via LC‐MS/MS in strains expressing either an empty vector control, *atu5372* or *atu2691*. Native DGSc were induced with 0.5 mM IPTG for 16 h in 523 rich medium and cells equivalent to an OD_600_ 5 were collected. Samples of interest were compared to a standard curve derived from measurements of known concentrations of pure cdi‐GMP to determine the concentration (in nM) of c‐di‐GMP in the samples. All experiments are the mean of two independent biological experiments with standard deviation error bars.

We then performed *in vitro* killing assays with each of the recombinant strains. Significant reductions in killing, comparable with what is observed with the *tssL* mutant, were seen upon expression of *dgcA* and *atu5372* while a reduced but not statistically significant killing was observed for strains expressing *atu2091* and *dgcC* (Fig. 4A). No reduction in killing was observed with strains expressing *atu1207* and *atu2691*. To confirm that the observed phenotypes were a direct result of altered levels of c‐di‐GMP, the levels of c‐di‐GMP were quantified in strains expressing either p*atu5372* or p*atu2691*. As expected, the strain expressing p*atu2691* did not display elevated levels of c‐di‐GMP compared to the vector control, while a strain expressing *atu5372* had levels of c‐di‐GMP higher than those seen in a strain expressing the known active *P. aeruginosa* DGC, *sadC* (Figs 4B and 1D). It was intriguing to observe that some of the DGCs tested were unable to impact the T6SS suggesting that strength in DGC activity might be an issue in signalling and that, such as with a rheostat, once a threshold of intracellular c‐di‐GMP is reached, some phenotypes could be triggered while others might need a higher threshold. This observation might also be in line with the established concept for the need of spatial pools of c‐di‐GMP (Christen *et al.*, 2010), and specific subcellular localization of some DGCs such as within the cytoplasmic membrane, or their direct interaction with the effector generating the output (McCarthy *et al.*, 2017a), would overcome the threshold issue.

### C‐di‐GMP regulates both the imp and hcp T6SS clusters at the transcriptional level independently of the ExoR signalling cascade

**Figure 5:**
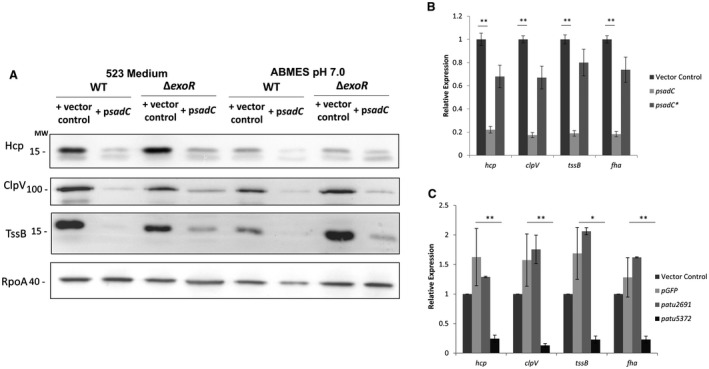
c‐di‐GMP impacts T6SS at the transcriptional level. A. Western blot using Hcp, TssB, ClpV, and RpoA (Loading control) antibodies on whole cell lysate of *ΔexoR* cells transformed with either Empty Vector control or p*sadC* grown to early exponential phase in 523 medium or AB‐MES pH7.0. B. qRT‐PCR on RNA isolated from cells transformed with either an empty vector control, p*sadC* or psadC* grown to early exponential phase. Expression quantified using primers specific to either *tssB*, *fha*, *hcp*, *clpV* and 16s rRNA which serves as a normalization control. Statistical significance was determined using students *t*‐test with p < 0.05 *, p < 0.01 **, p < 0.001*** comparing the vector control to p*sadC*. C. qRT‐PCR on RNA isolated from cells transformed with either an empty vector control, p*GFP*, p*atu2691* or p*atu5372* grown to early exponential phase. Expression quantified using primers specific to either *tssB*, *fha*, *hcp*, *clpV* or 16s rRNA which serves as a normalization control. Statistical significance was determined using students *t*‐test with p < 0.05 *, p < 0.01 **, p < 0.001*** comparing each strain to the expression control p*GFP*. All experiments are the mean of three independent biological experiments with standard deviation error bars.

Although T6SS regulation in *A. tumefaciens* is relatively poorly understood, one pathway involved is the ExoR/ChvI/ChvG signalling system which is known to impact EPS production, horizontal gene transfer, motility and virulence (Wu *et al.*, 2012; Heckel *et al.*, 2014). The ChvG/ChvI two‐component system positively regulates the T6SS while ExoR acts negatively and is a periplasmic repressor (Wu *et al.*, 2012). In acidic pH conditions such as those found around a plant wound site, ExoR is degraded, this allows the autophosphorylation of ChvG, which can then transfer a phosphoryl group to its cognate response regulator ChvI for activation of T6SS (Wu *et al.*, 2012) (see Discussion). To assess if the impact on T6SS through c‐di‐GMP signalling involves this cascade, p*sadC* was introduced into an *A. tumefaciens* *exoR* mutant which is known to have elevated activity of T6SS. If the elevated pool of c‐di‐GMP is acting through the ExoR cascade, then c‐di‐GMP transmission should be interrupted in the *exoR* mutant and no longer be able to impact on the T6SS. This was assessed in standard 523 medium, but also in minimal media (AB‐MES pH7), a condition where ExoR‐mediated repression of T6SS has previously been demonstrated (Wu *et al.*, 2012). Remarkably, it was observed that in the *exoR* mutant, expression of *sadC* and thus high levels of c‐di‐GMP are still capable of down‐regulating the T6SS, as seen by monitoring Hcp, ClpV and TssB production (Fig. 5A) suggesting that c‐di‐GMP is acting independently of the ExoR network. Since all data presented thus far of c‐di‐GMP impacting the T6SS were at the protein level, we also wanted to establish if this regulation was originally exerted at the transcriptional level. To address this, RNA was isolated from *A. tumefaciens* transformed with either the cloning vector, a vector expressing a nonspecific protein GFP, p*sadC*, psadC*, p*atu2691*, a proposed inactive DGC or p*atu5372*, a native *A. tumefaciens* DGC that was shown to have a significant impact on clumping, interspecies killing and Hcp secretion. qRT‐PCR was performed on cDNA synthesized using RNA extracted from these strains and primers specific to genes belonging to both divergent operons of the *A. tumefaciens* T6SS cluster, namely *tssB*, *fha*, *hcp* and *clpV* (Figs 1A and 5B,C). Remarkably, all of these genes were significantly down‐regulated when *atu5372* or *sadC* was expressed, but not upon expression of an inactive sadC*, a proposed inactive DGC *atu2691* or a nonspecific protein *GFP*, confirming that the impact of c‐di‐GMP on T6SS also occurs at the transcriptional level and that all T6SS genes are impacted.

### C‐di‐GMP negatively impacts expression of components of the T4SS and the ability of *A. tumefaciens* to transform plant cells

**Figure 6:**
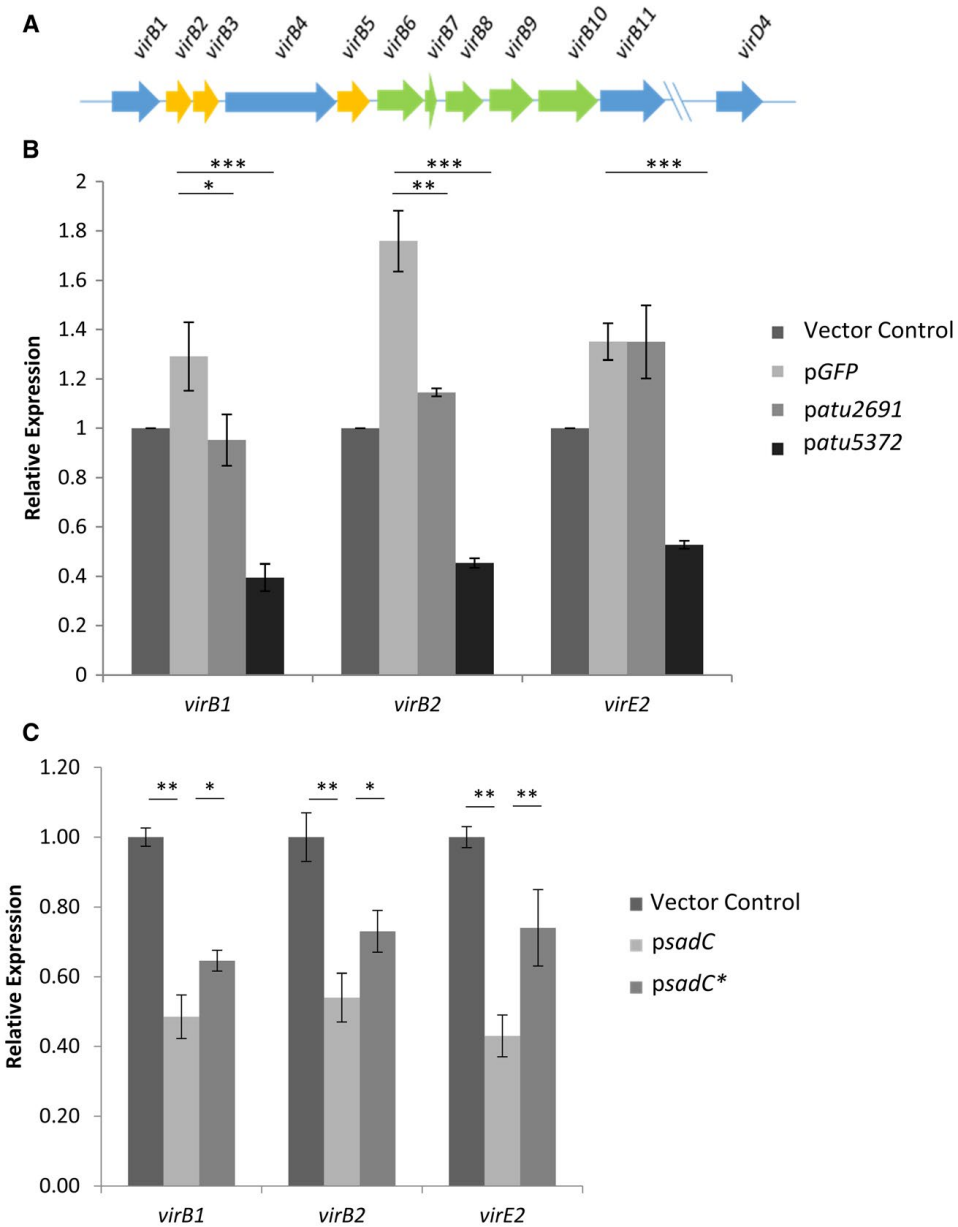
Impact of c‐di‐GMP on T4SS. A. Operon encoding components of the T4SS, *virB1‐11* represents the structural components of the T4SS machinery while *virD4* is involved in coupling DNA transfer. B. qRT‐PCR on RNA isolated from cells transformed with either an empty vector control, p*GFP*, p*atu2691* or p*atu5372* grown in AB‐MES medium (pH 5.5) containing 200 μM acetorysingone (AS) for virulence gene induction. Expression quantified using primers specific to either *virB1*, *virB2*, *virE2 *or 16s rRNA which serves as a normalization control. All experiments are the mean of three independent biological experiments with standard deviation error bars. Statistical significance was determined using students's *t*‐test with p < 0.05 *, p < 0.01 **, p < 0.001*** when compared to the protein expression control p*GFP*. C. qRT‐PCR on RNA isolated from cells transformed with either an empty vector control, p*sadC*, psadC* grown to early exponential phase. Expression quantified using primers specific to *virB1*, *virB2*, *virE2* and 16s RNA which serves as a normalization control. All experiments are the mean of three independent biological experiments with standard deviation error bars. Statistical significance was determined using students's *t*‐test with p < 0.05 *, p < 0.01 **, p < 0.001***.

The significant impact of c‐di‐GMP on the activity of the T6SS and biofilm formation led us to ask whether other determinants involved in infection progression, such as the T4SS, were also responsive to the intracellular levels of c‐di‐GMP. The rationale here was in line with previous reports where protein secretion systems, e.g. the T6SS and T3SS, are antagonistically controlled (Moscoso *et al.*, 2011; McCarthy *et al.*, 2017b). As described above for the control of EPS production, the regulation of the T4SS is dependent on activation of the VirA/VirG signalling by acidity or phenols such as acetosyringone (AS), in which the transcription of the gene encoding the VirG response regulator is activated *via* the ExoR/ChvI/ChvG signalling system (Heckel *et al.*, 2014). Activation of this cascade subsequently confers the ability of *A. tumefaciens* to transfer T‐DNA into the host plant cell. We thus analysed the expression of a number of different T4SS genes (*virB1*, *virB2*) and T4SS effector gene *virE2* in cells expressing the native DGCs (Fig. 6A). qRT‐PCR was performed on cDNA synthesized using RNA isolated from cells transformed with either a vector control, a vector expressing a nonspecific protein GFP, p*atu2691* or p*atu5372*. Our results suggested that increasing the levels of c‐di‐GMP within the cell significantly down‐regulates the expression of T4SS machinery and effector genes, while expression of p*atu2691*, a proposed inactive DGC or a non‐specific protein p*GFP* did not negatively impact expression (Fig. 6B). Similar results were seen when cells were expressing p*sadC*, although only partial but significant restoration of expression was seen when expressing psadC* (Fig. 6C).

**Figure 7:**
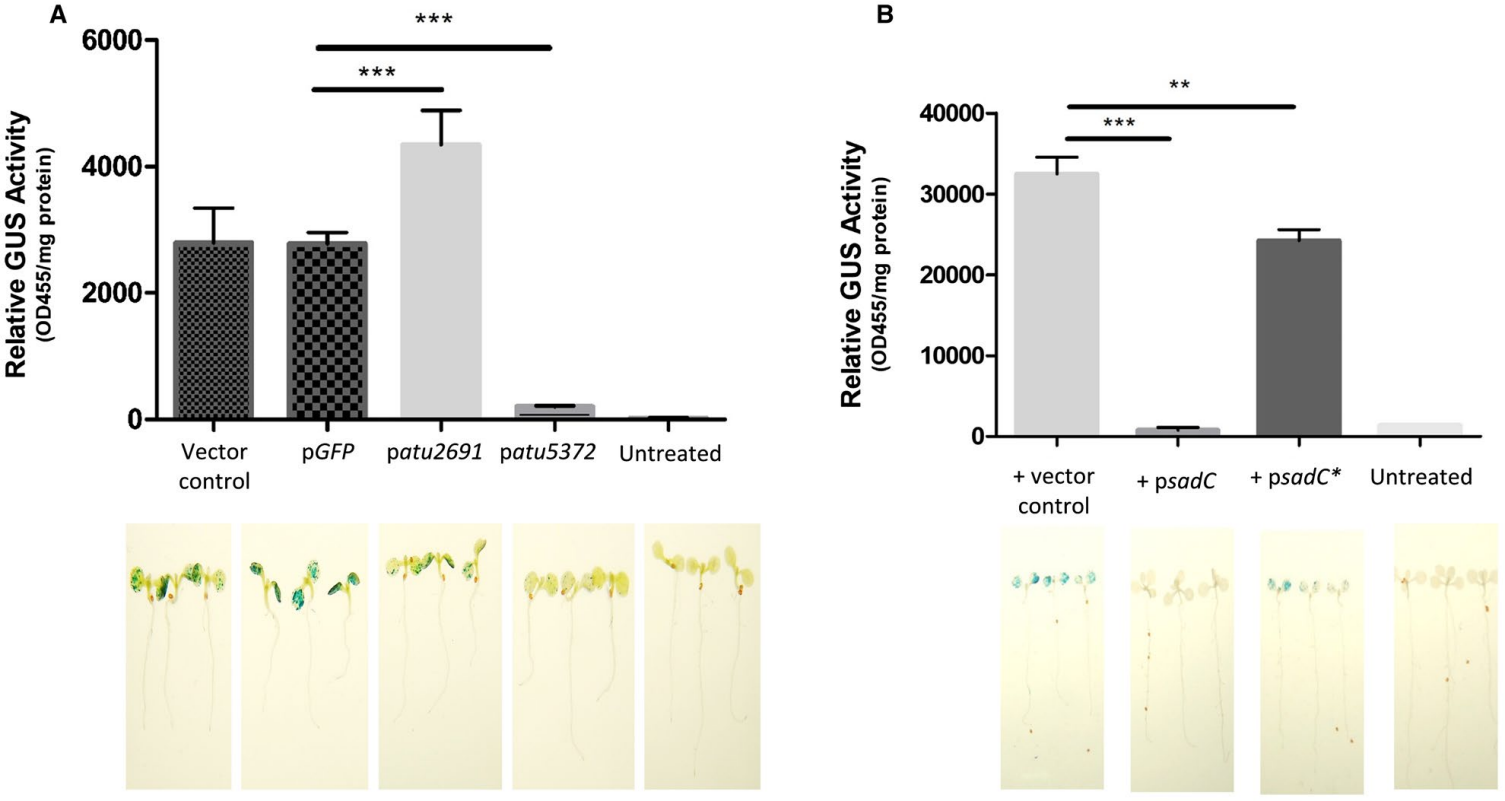
Impact of c‐di‐GMP on plant transformation. A. Four‐day‐old *Arabidopsis* seedlings were infected with *Agrobacterium* strain C58C1(pTiB6S3ΔT)^H^ carrying pBISN1 and either an empty vector control, p*GFP*, p*atu2691* or p*atu5372*. These cells were pre‐incubated in I medium (pH5.5) supplemented with 200 μM AS to induce expression of the *vir* genes. Seedlings were stained with 5‐bromo‐4‐chloro‐3‐indolyl glucuronide (X‐Gluc) to visualize the GUS activity. Statistical significance was determined using students's *t*‐test with p < 0.05 *, p < 0.01 **, p < 0.001*** when compared to the protein expression control p*GFP*. B. Four‐day‐old *Arabidopsis* seedlings were infected with *Agrobacterium* strain C58C1(pTiB6S3ΔT)^H^ carrying pBISN1 and either an empty vector control, p*sadC* or psadC*. These cells were pre‐incubated in I medium (pH5.5) supplemented with 200 μM AS to induce expression of the *vir* genes. Seedlings were stained with 5‐bromo‐4‐chloro‐3‐indolyl glucuronide (X‐Gluc) to visualize the GUS activity. All experiments are the mean of three independent biological experiments with standard deviation error bars. Statistical significance was determined using students's *t*‐test with p < 0.05 *, p < 0.01 **, p < 0.001*** when compared to the vector control.

To further interrogate this and determine if this impact on transcription is biologically relevant, an AGROBEST assay was performed (Wu *et al.*, 2014). This is an *Agrobacterium*‐mediated *Arabidopsis* transformation assay (*Agrobacterium*‐mediated enhanced seedling transformation) that utilizes β‐glucuronidase (GUS) as a reporter carried on the T‐DNA to determine the impact of native regulatory elements on *Agrobacterium* transformation activity (Wu *et al.*, 2014). Four‐day‐old *Arabidopsis* seedlings were infected with *A. tumefaciens* strain C58C1 (pTiB6S3ΔT)^H^ carrying either an empty vector control, a vector expressing a nonspecific protein GFP, p*atu2691* or p*atu5372* and expressing a GUS reporter. The assay was allowed to proceed for 3 days before seedlings were stained with 5‐bromo‐4‐chloro‐3‐indolyl glucuronide (X‐Gluc) to visualize the GUS staining. Remarkably, only expression of p*atu5372* leads to a dramatic reduction in GUS staining (Fig. 7A) indicating that, in concordance with the qRT‐PCR data, the activity of the T4SS is significantly impacted by the intracellular levels of c‐di‐GMP. Similar findings were observed when cells were expressing p*sadC*, although expression of psadC* could not completely abrogate this (Fig. 7B).

## Discussion

*Agrobacterium tumefaciens* is a phytopathogen capable of causing tumorigenesis in a wide variety of plant species through T‐DNA transfer *via* the T4SS (Gelvin, 2010). The start of this transfer process is centered on *A. tumefaciens* sensing characteristic changes in the rhizosphere such as a drop in pH and phytochemical release, indicative of a plant wound. Intriguingly, a drop in pH has also been shown to induce not only the T4SS but also the T6SS. The latter is central to *A. tumefaciens* outcompeting other bacteria within this niche whereas the former allows the unimpeded transfer of T‐DNA and eventual formation of a crown gall (Wu *et al.*, 2012). Other than low pH, the understanding of what factors regulate the activity of the T6SS in *A. tumefaciens* is relatively limited. In this study, we investigate the role of c‐di‐GMP in regulating T6SS and T4SS in *A. tumefaciens*. Previously characterized DGCs and PDEs from *P. aeruginosa* were transformed into *A. tumefaciens*. The membrane bound DGC called SadC (Merritt *et al.*, 2007a) was shown to significantly induce a clumping phenotype, characteristic of high levels of c‐di‐GMP (Heindl *et al.*, 2014) (Fig. 1B), and down‐regulate the expression of both the T6SS toxins and T6SS machinery components (Fig. 1C). This is contrary to the established paradigm in *P. aeruginosa* where high levels of c‐di‐GMP are associated with increased levels of T6SS (Supporting Information Fig. S2) (Moscoso *et al.*, 2011). We demonstrated that the ability to impact T6SS was dependent on SadC being active as a version of SadC that features a mutated GGDEF domain had no impact on bacterial clumping or T6SS activity (Fig. 1B and C). The biological relevance of this down‐regulation is validated by the inability of a strain of *A. tumefaciens* transformed with a plasmid expressing *sadC* to kill a prey bacterium *in vitro* or in the native environment of a plant (Fig. 2A and B).

The use of an active DGC from *P. aeruginosa* could create an artificial pseudo‐circuit inhibiting the activation of the T6SS. To rule this out and to explore the capacity of native *A. tumefaciens* DGCs to down‐regulate T6SS, the genes of six different *A. tumefaciens* DGCs, that have similar features as SadC, e.g. predicted transmembrane domains, were cloned and expressed. Three of these DGCs, DgcA, Atu2091 and Atu5372, were capable of down regulating the secretion of Hcp to non detectable levels, comparable to those observed in an *A. tumefaciens* T6SS mutant, *ΔtssL* (Fig. 3B). When further assessed, these DGCs were all capable of inhibiting interbacterial killing by *A. tumefaciens* both *in vitro* and *in planta* to differing degrees. These findings confirm the hypothesis that in *A. tumefaciens* high levels of c‐di‐GMP down‐regulate T6SS but they also offer an intriguing insight into the specificity exhibited by different DGCs. The differing levels of killing observed in strains expressing different DGCs (Fig. 4A) highlights the importance of c‐di‐GMP threshold levels to trigger specific output, and may also lend support to an emerging phenomenon whereby the spatial and temporal localization of a DGC can significantly influence the resulting phenotypic impact (Romling *et al.*, 2013; Dahlstrom and O'Toole, 2017).

**Figure 8:**
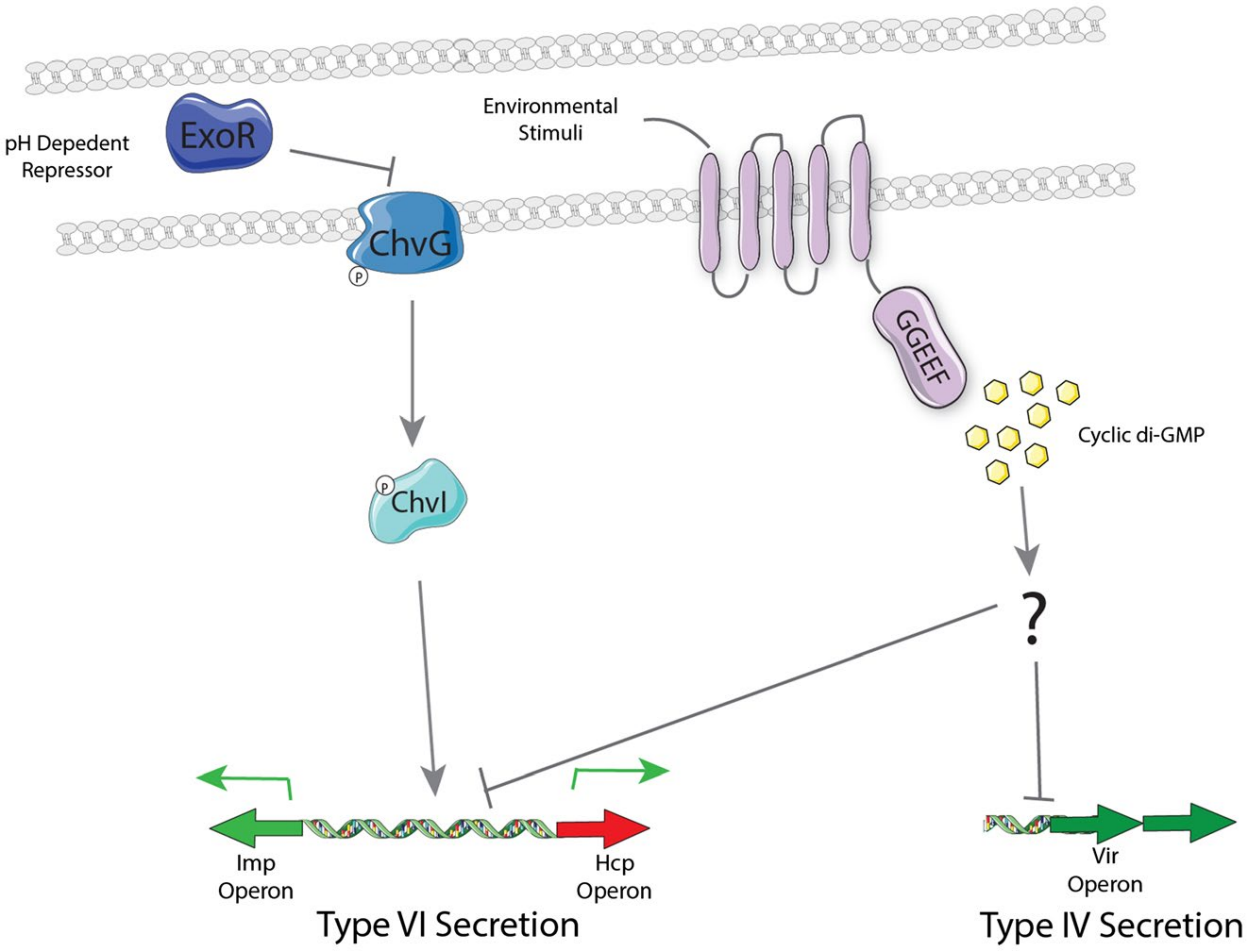
Regulation of T6SS and T4SS in *A. tumefaciens*. In acidic pH conditions such as those found around a plant wound site, the periplasmic regulator ExoR is degraded, this allows the activation of ChvG *via* autophosphorylation, ChvG can then activate its cognate response regulator ChvI, which then activates the T6SS. The activation of native DGCs by environmental stimuli leads to an increase in the local levels of cyclic di‐GMP. These increased levels of c‐di‐GMP are capable of repressing the transcription of the T6SS and the T4SS *via* an as yet uncharacterized regulator(s).

The regulation of the T6SS is shown to occur at the transcriptional level with both divergent transcriptional units in the T6SS cluster being significantly down‐regulated (Fig. 5B and C). The only characterized signalling cascade involved in the regulation of T6SS in *A. tumefaciens* is the ExoR/ChvI/ChvG system (Wu *et al.*, 2012; Heckel *et al.*, 2014); however, we collected data suggesting that the impact of c‐di‐GMP acts independently of this regulatory network (Fig. 5A). This finding prompted us to explore the influence of intracellular c‐di‐GMP levels on the T4SS, another ExoR/ChvI/ChvG target. Intriguingly, we observed a significant transcriptional down‐regulation in the expression of key components of the T4SS machinery when a DGC was expressed, *sadC* or *atu5372*, and this requires c‐di‐GMP synthesis since an inactive SadC, SadC*, displays only traces of inhibition of T‐DNA transfer into *Arabidopsis* plant cells (Fig. 7AB). Intriguingly, it seems here that c‐di‐GMP signalling is independent of the master regulator for T4SS and T6SS expression, i.e. ChvI/ChvG/ExoR. This is slightly different from what is observed with *P. aeruginosa*, in which the master network controlling T6SS activity, Gac/Rsm, is entangled with c‐di‐GMP signalling. In this case, the gene encoding the DGC SadC, is directly controlled by the translational repressor RsmA (Moscoso *et al.*, 2014), which in turn modulates c‐di‐GMP levels. Note that the absence of ExoR, relieves the kinase ChvG which can then phosphorylate the response regulator ChvI and therefore activate the T6SS and the T4SS genes. In this case if c‐di‐GMP was binding ChvI or ChvG to inhibit their activity, such inhibition should have been detected in an *exoR* mutant, which was not the case (Fig. 5A). Alternatively, a plausible explanation is that c‐di‐GMP could modulate the activity of another protein, e.g. a membrane protein interacting with ExoR. This protein could capture the periplasmic ExoR when it is not bound to c‐di‐GMP. Instead, when such an integral membrane protein is bound to c‐di‐GMP, interaction with ExoR does not occur and ExoR is free and available to inhibit the ChvI kinase (Fig. 8). This type of control at the interface cytoplasm/periplasm and across the cytoplasmic membrane was previously shown in the case of the *P. fluorescens* Lap system (Hinsa and O'Toole, 2006; Newell *et al.*, 2009). In this case, the degenerated LapD cyclase/phosphodiesterase is an integral cytoplasmic protein, which can bind c‐di‐GMP and then interact with the periplasmic LapG protease. When LapD is c‐di‐GMP‐bound, it prevents the LapG protease from cleaving and releasing the surface adhesin LapA, which contributes to biofilm formation only when it remains associated with the cell surface (Navarro *et al.*, 2011; Dahlstrom and O'Toole, 2017). This is in agreement with the dogma‐high c‐di‐GMP/high biofilm. Future work could focus on identifying possible missing links such as the one suggested above. Interestingly the active cyclases are membrane embedded, such as SadC in contrast to WspR, and that may reinforce the idea that the signalling could be a transmembrane inside‐out signalling, where the membrane cyclase or an interacting partner therein plays a key role in the process (Navarro *et al.*, 2011). In the inside‐out signalling mechanism described by the O'Toole laboratory, the transmission of the signal involves HAMP domains so a directed approach could be used to assess known c‐di‐GMP‐related proteins with a HAMP domain (Navarro *et al.*, 2011). Alternatively a non‐biased approach using random transposon mutagenesis in a strain overexpressing a DGC and carrying a reporter fusion for T6SS or T4SS expression could be used. Screens could be aimed at identifying mutants whose T4SS or T6SS activity goes high up even in the context of DGC expression, thus pinpointing any players in the circuitry. In any case, c‐di‐GMP signalling has shown that the combination and sequence of events that lead from synthesis to output is far from a simple and stereotyped trajectory. Examples from within *A. tumefaciens* have shown that a dual DGC‐PDE, called DcpA, can see its activity balanced from DGC to PDE based on its interaction with another protein called PruA, a pterin reductase. In the presence of PruA, DcpA is a PDE while in its absence it is a DGC. This implies that when DcpA is expressed in a heterologous host, such as *E. coli* in which PruA is absent, the main activity is PDE, while in *Agrobacterium* it is mostly a DGC (Feirer *et al.*, 2015). This has also lended support to our rationale to analyse the impact of native *Agrobacterium* DGC versus heterologous ones from *P. aeruginosa* to fully validate our phenotypic observations.

In *P. aeruginosa*, the switch in lifestyle from biofilm to motile is also tightly linked to a concomittant switch from T6SS to T3SS respectively (Moscoso *et al.*, 2011; Valentini and Filloux, 2016; McCarthy *et al.*, 2017b). This makes sense if considering that the T6SS may help killing unwanted members of a biofilm population and instead prefering the T3SS cytotoxicty when adopting an acute infectious style. In the case of *Agrobacterium*, simultaneous down‐regulation of the T6SS and T4SS when biofilm formation is induced gives an intriguing insight into the phases of *A. tumefaciens* infection. The upregulation of motility genes, the T6SS and the T4SS by low pH and phytochemical release associated with a plant wound is the first step in *A. tumefaciens* targeting the wound site and ensuring a mono‐infection by killing competing species (Merritt *et al.*, 2007b; Wu *et al.*, 2012). Once at the site of the wound it is possible that intracellular levels of c‐di‐GMP are elevated stimulating the production of EPS, attachment and biofilm formation (Heindl *et al.*, 2014). T6SS and T4SS are concurrently downregulated possibly as an economy measure allowing more efficient channelling of resources towards establishing infection at the site of the wound. Once initial sustainable colonization is established, variation in c‐di‐GMP levels can then allow further colonization and again competition with other bacteria and modification of the plant cell environment. The orchestration of c‐di‐GMP levels and its variation in different cells and at a different localization in each cell, would be in accordance with a model, where the spatio‐temporal dynamics of c‐di‐GMP is key (Christen *et al.*, 2010; Abel *et al.*, 2013; Kulasekara *et al.*, 2013; Skotnicka *et al.*, 2016). It will be instrumental to the fate of the community at a population level but may of course be specific to some individuals, offsprings of which will constitute a new sub‐population with dictinct aims, such as, for example, dispersal from the biofilm. Changes in c‐di‐GMP within a cell have been demonstrated on numerous instances to significantly alter the behavior of that cell, examples of this include the temporally oscilating global pools of c‐di‐GMP in *Caulobacter crescentus* which play a key role in regulating the swarmer to stalked cell transition and in *Myxococcus xanthus* where flucatuations in c‐di‐GMP levels control the developmental cycle that results in fruiting body formation (Abel *et al.*, 2013; Kulasekara *et al.*, 2013; Ozaki *et al.*, 2014; Lori *et al.*, 2015; Rotem *et al.*, 2015; Skotnicka *et al.*, 2016).

The impact seen in *A. tumefaciens* is to down‐regulate both T4SS and T6SS, so this duality may have significant impact on shaping future bio‐control efforts in the agri‐sector. Exploiting the knowledge that both the T4SS and the T6SS are down regulated in the presence of elevated levels of c‐di‐GMP (Fig. 8) may also prompt the development of exogenous soil treatments that could influence the accumulation of this signalling molecule within the cell and in turn influence its physiology/pathogenicity. Similar approaches have proven successful in disrupting *P. aeruginosa* biofilms in the cystic fibrosis lung, whereby exposure of the lung to nitric oxide induces biofilm dispersal through the modulation of the activity of phosphodiesterases, thus manipulating the intracellular levels of c‐di‐GMP (Barraud *et al.*, 2006; Barraud *et al.*, 2009; Howlin *et al.*, 2017). In all, while the hypothesis that c‐di‐GMP is fine tuning the behaviour of *A. tumefaciens* as it transitions from the rhizosphere to the plant surface remains to be verified, here we have demonstrated that c‐di‐GMP is capable of significantly influencing the ability of *A. tumefaciens* to compete with other bacterial species and thus may shape the microbial diversity within the soil and also significantly impacts the capacity of *A. tumefaciens* to carry out one of its most defining features, the ability to transform the plant cell with interkingdom DNA transfer.

## Experimental procedures

### Bacterial strains and plasmids

Strains, plasmids and primer sequences used in this study are shown in Supporting Information Table [Link], [Link] and [Link], [Link]. *Escherichia coli* was cultured in Lysogeny Broth, whereas 523 medium (Kado and Heskett, 1970) was routinely used for *A. tumefaciens* strains unless otherwise indicated. Growth conditions were previously described (Ma *et al.*, 2009). When required, appropriate antibiotics were added to the medium as follows: for *E. coli*, 50 µg ml^−1^ ampicillin, 50 µg ml^−1^ gentamicin (Gm), 50 µg ml^−1^ kanamycin (Km) and 100 µg ml^−1^ spectinomycin; and for *A. tumefaciens*, 200 µg ml^−1^ spectinomycin. About 1mM Isopropyl‐β‐D‐thiogalactopyranoside (IPTG) was used when necessary. Site directed mutagenesis was performed on pBBRMCS4‐*sadC* using primers (Supporting Information Table S1) targeted to the catalytic site GGDEF site with specific nucleotide changes corresponding to mutation of the active site to AAAEF. These primers were used to amplify the full plasmid, followed by ligation with T4 ligase and subsequent transformation into *E. coli* DH5α. Congo red assays were performed as previously described (Moscoso *et al.*, 2011).

### Interbacterial competition on agar plates

Interbacterial competition assays were performed as previously described (Ma *et al.*, 2014; Bondage *et al.*, 2016). In brief, overnight cultures of *E. coli* DH10B containing pRL662 derivative conferring gentamicin resistance were grown in LB at 37°C. The *A. tumefaciens* cells were adjusted to OD_600_ 0.1, whereas the *E. coli* DH10B were adjusted to OD_600_ 0.01, mixed at a 10:1 ratio, and 10 μl was spotted on LB (pH7.0) agar and incubated for 16 h at 28°C. Cells were harvested, serially diluted, and plated in triplicates on LB agar with or without gentamicin for colony forming units (CFU) counting. All experiments are the mean of a minimum of three independent biological experiments with standard deviation error bars. Statistical significance was determined using students *t*‐test with p < 0.05 *, p < 0.01 **. Bacterial competitions with *P. aeruginosa* were carried out as described before (Pissaridou *et al.*, 2018). Briefly, strains harbouring indicated plasmids were grown over night with appropriate antibiotics and competition assays were performed the next day on LB agar plates without antibiotics using a 1:1 ratio of attacker to prey and incubating at 37°C for 24 h. Attacker strains were *P. aeruginosa* PAO1 and PAO1 Δ*rsmA* and prey strain was *E. coli* Top10 pRL662‐gfp. Competitions were recovered and serially diluted prior to spot plating on LB agar plates with antibiotics for selection and grown overnight at 37°C. Survival was assessed by quantitative colony counts on selective media (using gentamicin for selection of *E. coli* and ampicillin for *P. aeruginosa*).

### Measurement of c‐di‐GMP levels

For c‐di‐GMP quantification, samples were prepared as described previously (Valentini *et al.*, 2016) and analysed by liquid‐chromatography mass spectrometry (LC‐MS/MS). In brief, *A. tumefaciens* C58 strains harbouring pBBRMCS4 vectors constitutively expressing DGCs were grown overnight in 20 mL 523 medium and cells equivalent to an OD_600 _5 were collected by centrifugation. Strains harbouring pTrC200 vector or derivatives were grown overnight, adjusted to an OD_600_ 0.1 and grown to an OD_600_ 0.7. Native DGCs were induced with 0.5 mM IPTG for 16 h and cells equivalent to an OD_600_ 5 were collected. Collected cells were resuspended in extraction solution (Acetonitrile/methanol/water, 2/2/1, v/v/v), incubated on ice for 15 min and heated for 10 min at 95–99°C. Cells were centrifuged for 10 min at 4°C, 20,800 x g and supernatant fluid was collected. Extraction was repeated twice and supernatant fluids of the 3 extraction steps were combined and incubated at −20°C overnight. Extraction fluids were centrifuged again and supernatant fluid was analysed at the BIOLOG Life Science Institute (Biolog, Bremen) *via* LC‐MS/MS. Samples of interest were compared to a standard curve derived from measurements of known concentrations of pure c‐di‐GMP to determine the concentration (in nM) of c‐di‐GMP in the samples.

### Interbacterial competition assay *in planta*

Interbacterial competition assays *in planta* were performed as previously described (Ma *et al.*, 2014). The intra‐species *A. tumefaciens* competition assay was performed with a 10:1 attacker‐to‐target ratio by leaf infiltration of *N. benthamiana*. Briefly, 523 overnight‐cultured *A. tumefaciens* cells were sub‐cultured at 28°C in the same medium for further growth to OD_600_ 1.0–1.5. The harvested cells were resuspended in 1/2 Murashige and Skoog (MS) medium (pH 5.5) to an appropriate OD_600_. The attacker (OD_600_ 5) and target, *E. coli* DH10B containing pRL662 (OD_600_ 0.5) were mixed equally before infiltration into 2‐month‐old leaves of *N. benthamiana* with use of a needleless syringe. After 24‐h incubation at room temperature, the infiltrated spot was punched out, ground in 0.9% NaCl, serially diluted, and plated in triplicates on LB agar containing appropriate antibiotic to select for the target cells. All experiments are the mean of three independent biological experiments with standard deviation error bars. Statistical significance was determined using students *t*‐test with p < 0.05 *, p < 0.01 **.

### Agrobacterium infection in *Arabidopsis* seedlings and GUS activity assay

The procedures for the seedling transient transformation assay were adapted from (Kim *et al.*, 2006; Wu *et al.*, 2014) with modifications. *Agrobacterium tumefaciens* cells were pre‐induced by addition of 200 μM of acetosyringone at 28°C for 16 h and resuspended in appropriate buffer with cell density OD_600_ 0.02. Four‐day‐old *A. thaliana* seedlings were then incubated in 1ml of the buffer in a well of a 6‐well plate for another 3 days. The seedlings were then removed from the buffer for GUS staining or GUS activity assay. For GUS staining, seedlings were stained with 5‐bromo‐4‐chloro‐3‐indolyl glucuronide (X‐Gluc) at 37°C for 6 h as described. GUS activity assay was determined by the conversion of 4‐methylumbelliferyl‐β‐D‐glucuronide (4‐MUG) to 4‐methylumbelliferone (4‐MU). 4‐MU fluorescence (excitation. 356 nm, emission. 455 nm) was measured with a 96 microtiter‐plate reader (Bio‐Tek Synergy Mx) and amount of protein in the reaction was determined by a Bradford assay. The relative GUS activity was expressed as OD_455_ μg^−1^ protein (Wu *et al.*, 2014).

### RNA extraction and quantitative RT‐PCR

Strains were grown to early exponential phase before cells were pelleted. Total RNA was extracted from pellets by Total RNA Extraction Kit (Arrowtech). First‐strand cDNA was synthesized from 4 μg of total RNA with SuperScript III Reverse Transcriptase (Invitrogen) and random primers. Quantitative PCR were performed in QuantStudio 12 K Flex Real‐Time PCR machine (Applied Biosystems) with Power SYBR Green PCR Master Mix (Invitrogen).

### Production of T6SS components and T6SS‐dependent secretion assay

The T6SS components and effectors in whole cell and during secretion assays were performed as described previously (Lin *et al.*, 2014). Strains were grown to early exponential phase in both AB‐MES pH7 medium and 523 medium. Whole cell extract and secreted proteins were separated by SDS/PAGE and transferred onto a PVDF or nitrocellulose membrane using a transfer apparatus (Bio‐Rad). The membrane was probed with primary antibody against Tde1 epitope (Ma *et al.*, 2014), Hcp (Wu *et al.*, 2008), Tae (Ma *et al.*, 2014), ClpV (Wu *et al.*, 2012) and RpoA (Lin *et al.*, 2014) was used as an internal control then incubated with horseradish peroxidase‐conjugated anti‐rabbit secondary antibody (1:20,000) and visualized with the ECL system (Perkin‐Elmer).

## Author contributions

RMC, AF, MY, EML designed research. RMC, MY, KG, YCW performed research. RMC, AF, MY, EML analysed data. RMC and AF wrote manuscript.

## Data Availability

The data that support the findings of this study are available from the corresponding author upon reasonable request.
