# Supplementary material for: Cyclic di‐GMP inactivates T6SS and T4SS activity in Agrobacterium tumefaciens
Source: Mol Microbiol. 2019 Jun 4;112(2):632–48. doi: 10.1111/mmi.14279 (PMC6771610; doi:10.1111/mmi.14279)
Supplement: Supplementary file 1 [file MMI-112-632-s001.pptx]

## Slide 1
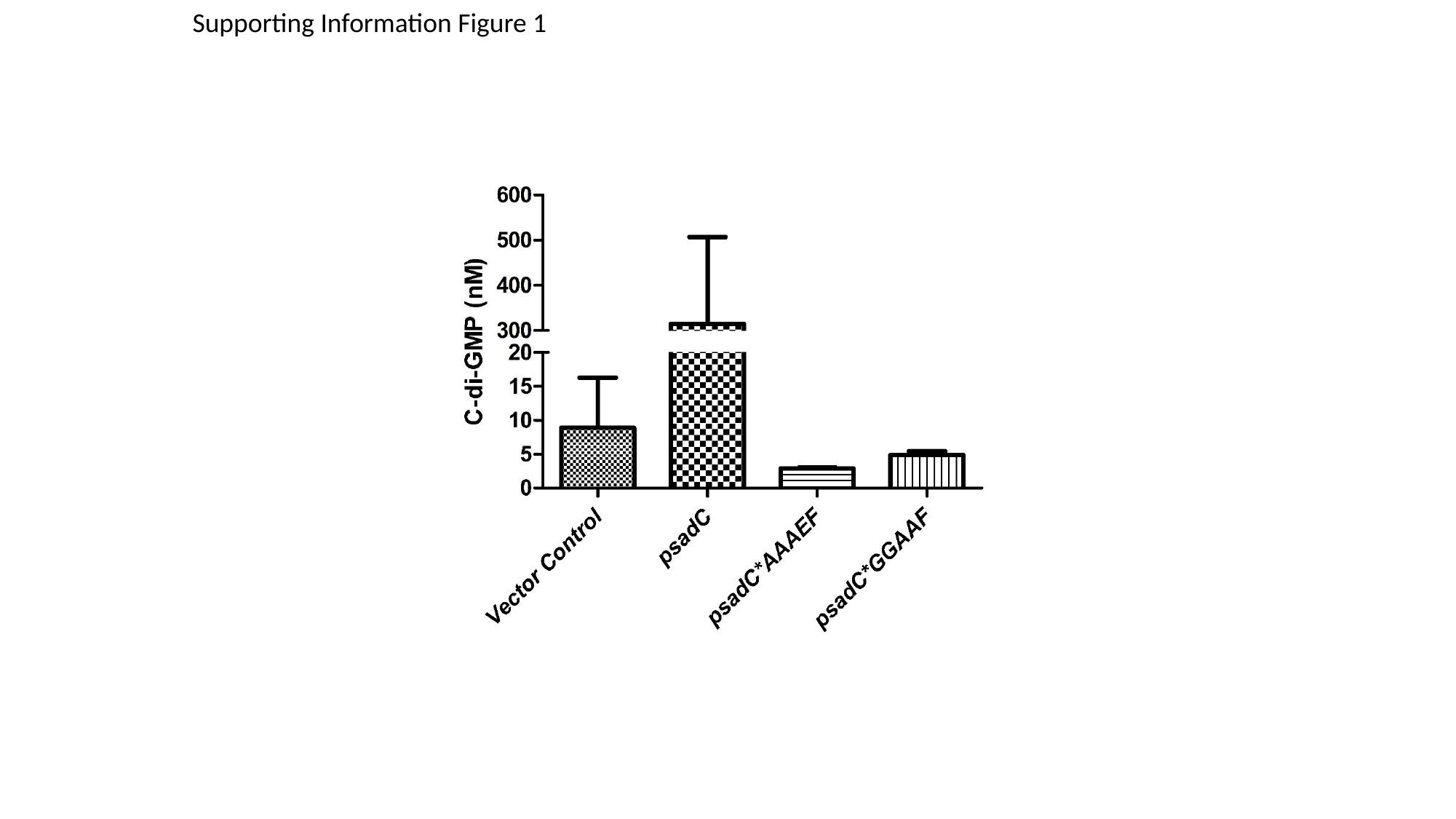

Supporting Information Figure 1

## Slide 2
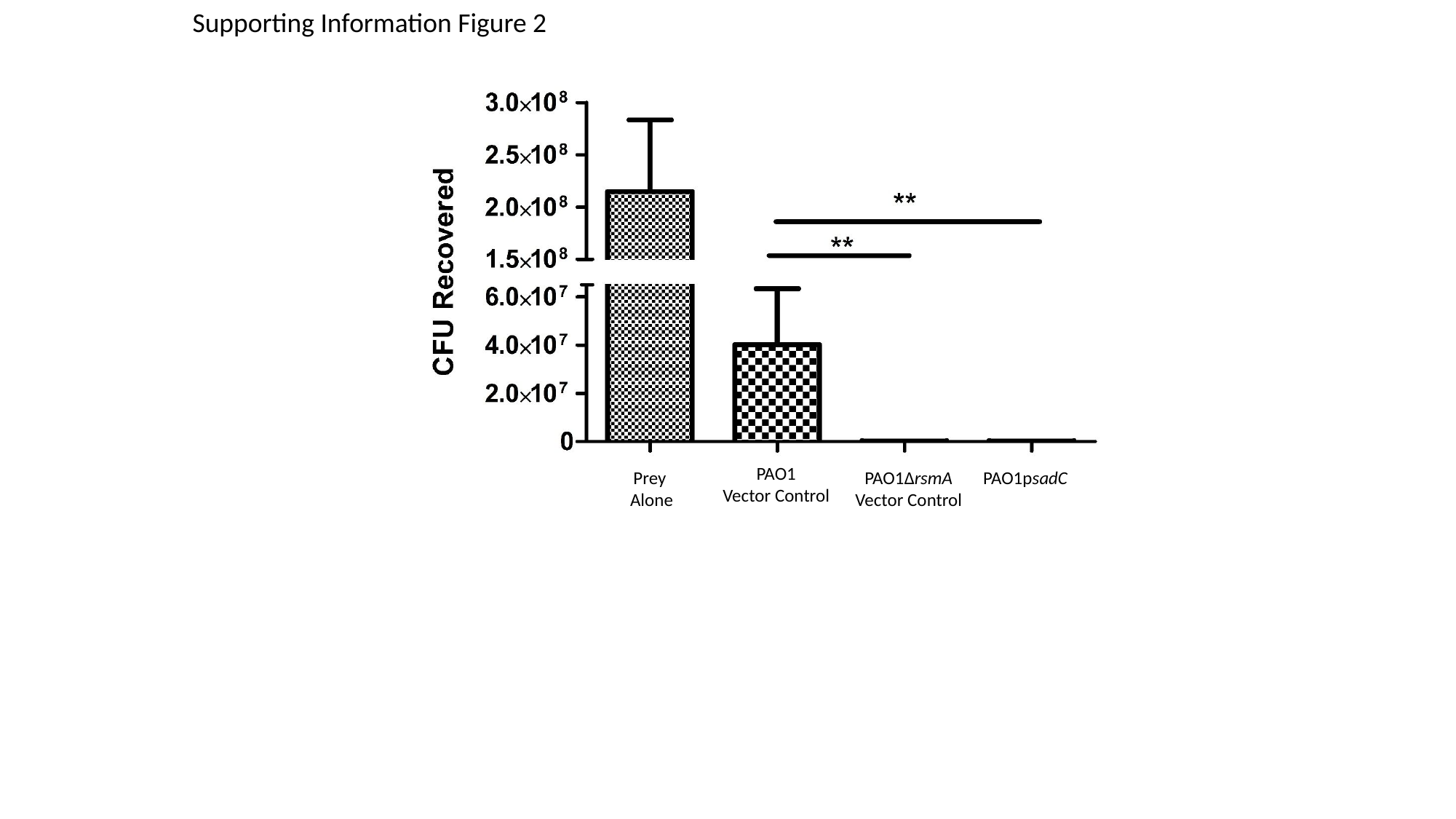

Supporting Information Figure 2
PAO1
Vector Control
Prey
Alone
PAO1ΔrsmA
Vector Control
PAO1psadC

## Slide 3
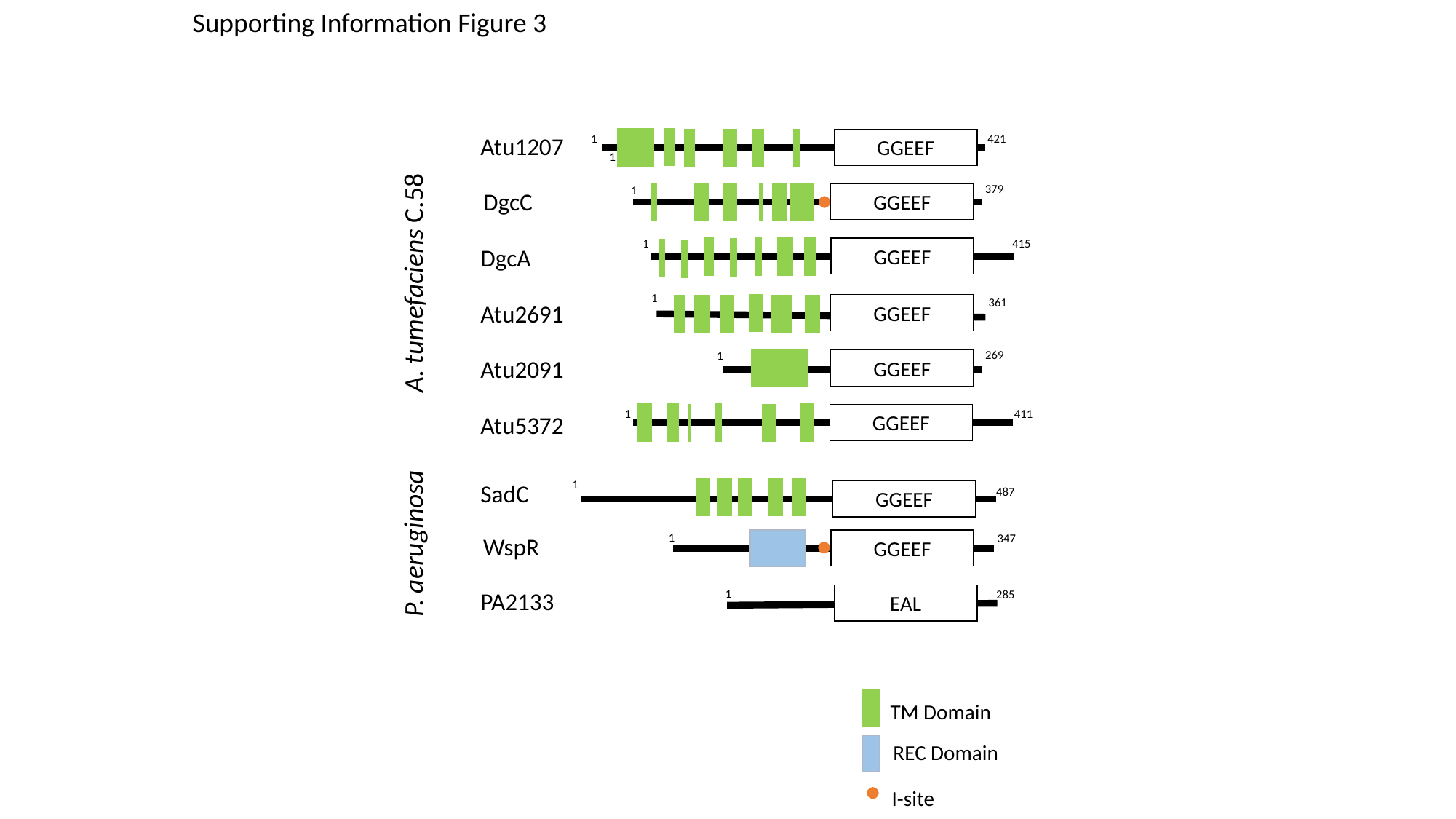

Supporting Information Figure 3
1
Atu1207
421
GGEEF
1
379
1
DgcC
GGEEF
1
415
DgcA
GGEEF
A. tumefaciens C.58
1
361
Atu2691
GGEEF
269
1
Atu2091
GGEEF
1
411
GGEEF
Atu5372
1
SadC
487
GGEEF
P. aeruginosa
1
347
WspR
GGEEF
1
PA2133
285
EAL
TM Domain
REC Domain
I-site
