# Supplementary material for: Cyclic di‐GMP inactivates T6SS and T4SS activity in Agrobacterium tumefaciens
Source: Mol Microbiol. 2019 Jun 4;112(2):632–48. doi: 10.1111/mmi.14279 (PMC6771610; doi:10.1111/mmi.14279)
Supplement: Supplementary file 2 [file MMI-112-632-s002.docx]

**Supplementary Table 1:** Primers used in this study
