# Supplementary material for: Cyclic di‐GMP inactivates T6SS and T4SS activity in Agrobacterium tumefaciens
Source: Mol Microbiol. 2019 Jun 4;112(2):632–48. doi: 10.1111/mmi.14279 (PMC6771610; doi:10.1111/mmi.14279)
Supplement: Supplementary file 3 [file MMI-112-632-s003.docx]

**Supplementary Table 2:** Strains and plasmids used in this study.

Hamilton RH, Fall MZ. 1971. The loss of tumor-initiating ability in *Agrobacterium* *tumefaciens* by incubation at high temperature. Experientia 27:229–230. doi:10.1007/BF02145913.

Moscoso JA, Mikkelsen H, Heeb S, Williams P, Filloux A. 2011. The *Pseudomonas aeruginosa* sensor RetS switches type III and type VI secretion via c-di-GMP signalling. Environ Microbiol.;13(12):3128-38.

Moscoso, J.A., Jaeger, T., Valentini, M., Hui, K., Jenal, U. and Filloux, A. 2014. The diguanylate cyclase SadC is a central player in Gac/Rsm‐mediated biofilm formation in *Pseudomonas aeruginosa*. *J. Bacteriol.* **196**, 4081–4088.

Vergunst AC, Schrammeijer B, den Dulk-Ras A, de Vlaam CM, Regensburg-Tuïnk TJ, Hooykaas PJ .2000 . VirB/D4-dependent protein translocation from *Agrobacterium* into plant cells. Science 290(5493):979-982.

Schmidt-Eisenlohr H, Domke N, & Baron C (1999) TraC of IncN plasmid pKM101 associates with membranes and extracellular high-molecular-weight structures in *Escherichia coli*. Journal of Bacteriology , 181 (18) 5563-5571;
